# Sight-Threatening Unilateral Posterior Scleritis With Positive Atypical p-ANCA As Early Manifestation of Lupus Spectrum Disease

**DOI:** 10.7759/cureus.58507

**Published:** 2024-04-18

**Authors:** Li Lian Chew, Wendy See, Chai Lee Tan, Shelina Oli Mohamed, Tajunisah Iqbal, Nazirah Ibrahim

**Affiliations:** 1 Department of Ophthalmology, Hospital Kuala Lumpur, Kuala Lumpur, MYS; 2 Department of Ophthalmology, Hospital Selayang, Selangor, MYS; 3 Department of Ophthalmology, Universiti Sains Malaysia School of Medical Sciences, Kota Bharu, MYS; 4 Department of Ophthalmology, Faculty of Medicine, Universiti Teknologi MARA, Cawangan Selangor, Kampus Sungai Buloh, Kuala Lumpur, MYS; 5 Department of Ophthalmology, Faculty of Medicine, University of Malaya Eye Research Centre, Universiti Malaya, Kuala Lumpur, MYS; 6 Department of Ophthalmology, Hospital Ampang, Selangor, MYS

**Keywords:** lupus spectrum disease, systemic lupus erythematosus, antinuclear cytoplasmic antibody, scleritis, atypical p-anca

## Abstract

Antinuclear cytoplasmic antibody (ANCA)-related scleritis is a potentially sight-threatening inflammatory condition that may occur as a primary vasculitis disorder or as a secondary vasculitis in a variety of inflammatory conditions. While ANCA has been classically associated with primary vasculitis diseases such as granulomatosis with polyangiitis (GPA), microscopic polyarteritis (MPA), and eosinophilic granulomatosis with polyangiitis (EGPA), it is interesting that in cases of lupus spectrum disease (LSD), both ANCA and atypical p-ANCA have been observed as secondary autoantibodies. Scleritis is a rare ocular manifestation of lupus disease with an incidence of around 1%.

This paper describes a case of sight-threatening posterior scleritis with positive atypical p-ANCA as an early manifestation of LSD. LSD is an acknowledged condition but frequently presents a diagnostic challenge or delay due to its ambiguous symptoms which may not fully align with the classification criteria of established systemic lupus erythematosus (SLE). Nonetheless, this condition should not be underestimated due to its potential impact on major organ involvement and its tendency to progress to established SLE. The diagnosis of LSD heavily relies on clinician suspicion, considering factors such as symptoms present in at least one organ system, positivity of antinuclear antibody (ANA), and clinical suspicion of future SLE development. Early identification allows for early treatment which would benefit high-risk patients.

A middle-aged Chinese lady presented with bilaterally asymmetrical eye redness and swelling, which was worse on the right side. Clinical examination revealed right eye proptosis, conjunctival injection, chemosis, scleral redness and binocular diplopia in all gazes. Right eye fundoscopic examination displayed extensive choroidal folds with a positive T-sign on the B-scan. Apart from ocular symptoms, there was no significant medical history related to autoimmune or connective tissue disorders. Her p-ANCA and c-ANCA results were negative, however atypical p-ANCA titer was positive with a high antinuclear antibody (ANA) titer of 1:1280 with a homogenous pattern. Additionally, she has a family history of systemic lupus erythematosus in her daughter. A diagnosis of right eye posterior scleritis secondary to underlying LSD was made. The scleritis was successfully treated with a combination of corticosteroid and systemic immunosuppressants and the patient was initiated on oral hydroxychloroquine to manage underlying LSD.

We aim to highlight to clinicians the diagnostic challenges associated with scleritis in LSD and emphasize the importance of prompt and timely multidisciplinary management in minimizing patient mortality and morbidity, as reflected in this case. This case of a positive atypical p-ANCA scleritis in LSD serves as an excellent example of effective management.

## Introduction

Scleritis is a chronic inflammatory condition that is characterized by edema and cellular infiltration of the sclera and episcleral tissue due to vasculitis of vessels supplying the coat of the eye [1,2]. Scleritis can occur in isolation or as a manifestation associated to systemic vasculitis, with nearly 50% of scleritis patients being associated with immune-mediated diseases [3]. Systemic vasculitis can be classified into primary vasculitis disease and secondary vasculitis. Primary vasculitis diseases include granulomatosis with polyangiitis (GPA), microscopic polyarteritis (MPA), eosinophilic granulomatosis with polyangiitis (EGPA), systemic lupus erythematosus (SLE), and rheumatoid arthritis (RA). Whereas, secondary vasculitis can develop later in the course of inflammatory disease and may be caused by various factors including infection, malignancies, and autoimmune diseases like inflammatory bowel disease and psoriatic arthritis [4].

The presence of antineutrophil cytoplasmic antibodies (ANCA) has been classically shown to be associated with primary vasculitis diseases such as granulomatosis with polyangiitis (GPA), microscopic polyarteritis (MPA), and eosinophilic granulomatosis with polyangiitis (EGPA). Interestingly, ANCA has also been reported as a secondary autoantibody in lupus patients, where both ANCA and atypical p-ANCA have been observed [5]. Scleritis is a rare ocular manifestation of lupus and occurs in approximately 1% of cases. This paper presents a case of sight-threatening posterior scleritis in a patient with positive atypical p-ANCA as part of the manifestation of lupus spectrum disorder (LSD.) LSD is a prevalent condition that can present as a diagnostic challenge or encounter diagnostic delay due to its ambiguous symptoms, which do not fully meet the classification criteria for established systemic lupus erythematosus (SLE) [6].

## Case presentation

A 41-year-old Chinese lady, diagnosed with type two diabetes mellitus one year ago, well-controlled with the latest HbA1c of 6.5%, presented with complaints of bilateral progressive worsening of eye redness and swelling, worse on the right eye for two months. It was associated with binocular diplopia and pain upon eye movement. There was no blurring of vision. The patient also denied experiencing constitutional or autoimmune symptoms such as fatigue, loss of weight, loss of appetite, hair loss, joint pain, ulcers, skin rash or discoloration, abdominal pain, and changes in bowel habits. However, family history revealed her daughter has active systemic lupus erythematosus (SLE) on treatment.

**Figure 1:**
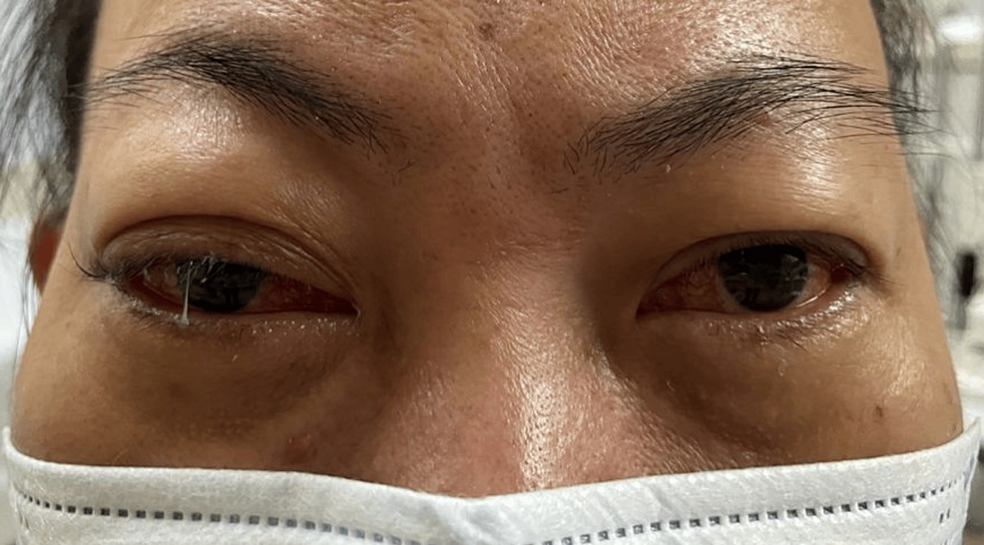
Bilateral lid fullness and conjunctival injection with right eye proptosis and chemosis.

**Figure 2:**
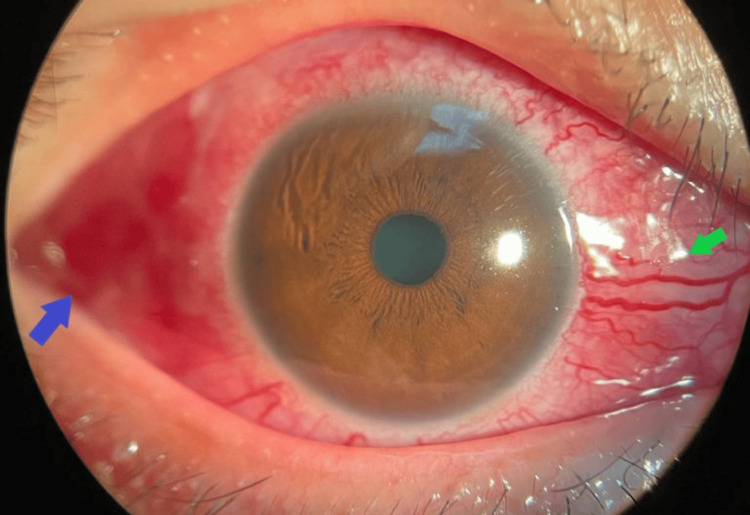
Right eye diffuse conjunctival injection with scleral redness shown with the blue arrow. Conjunctival chemosis and prominent episcleral vessels shown with the green arrow.

**Figure 3:**
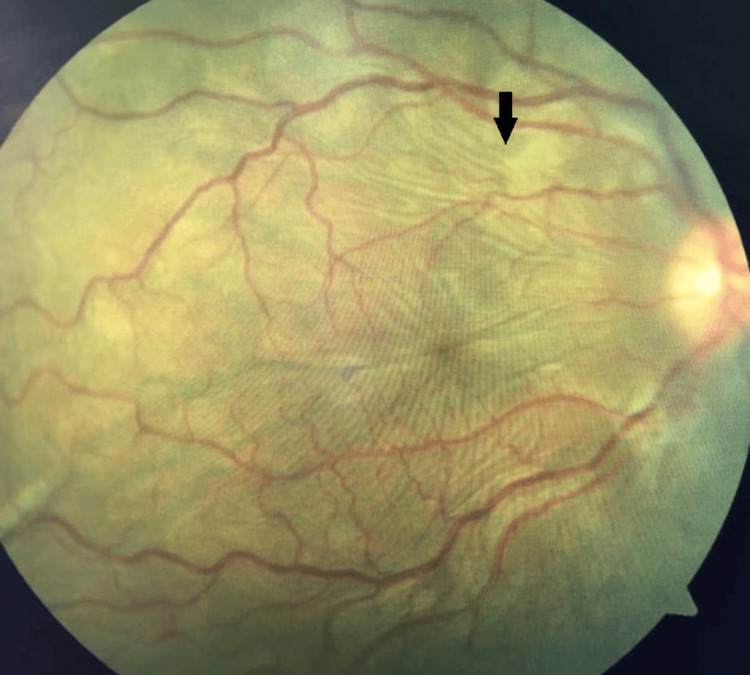
Right eye fundus demonstrating extensive choroidal folds highlighted with the black arrow.

**Figure 4:**
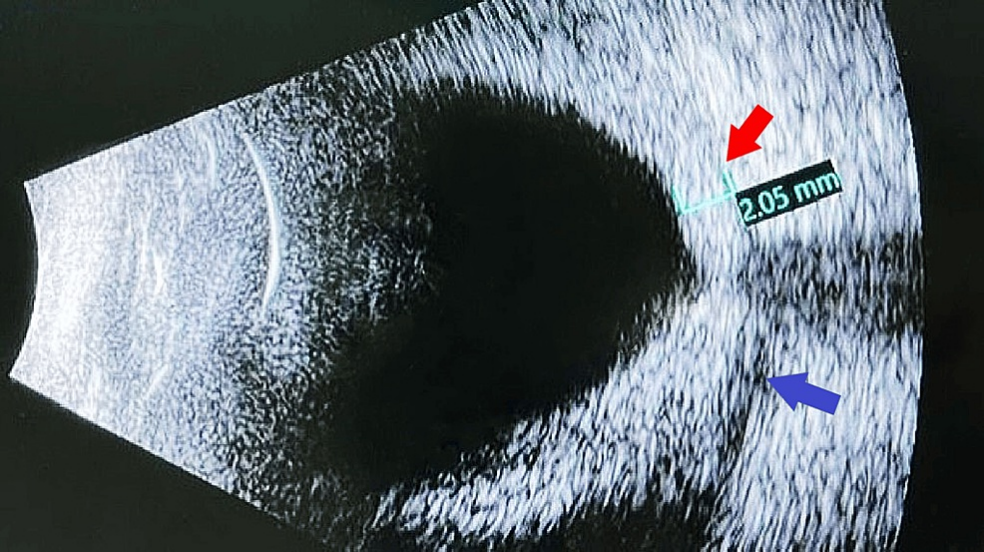
B-scan demonstrating thickened sclera highlighted with the red arrow and sub-tenon fluid collection posteriorly (positive T sign) highlighted with the blue arrow.

Ocular examination revealed bilateral lid fullness and conjunctival injection with right eye proptosis and chemosis, as shown in Figure 1. The patient’s presenting Snellen visual acuity was 6/24 and 6/18 on the right and left eye respectively. There was no observed relative efferent pupillary defect. However, there was a reduction in perception of light brightness and red desaturation in the right eye, to about 70 percent. The Ishihara test and confrontation test for both eyes were otherwise normal. Ocular motility showed limitation of right eye elevation with the presence of binocular diplopia in all directions of gazes. The right eye showed proptosis of 17mm, whereas the left eye measured 15mm on the Hertel exophthalmometer. Anterior segment examination revealed bilateral diffuse conjunctival injection with prominent episcleral vessels, with more pronounced scleral redness observed in the right eye. Additionally, the right eye exhibited chemosis, as shown in Figure 2. The intraocular pressure in the right eye ranges between 22 and 30 mmHg, while in the left eye, it ranges from 20 to 22 mmHg. Fundus examination revealed extensive choroidal folds on the right eye with normal optic nerve appearance, as shown in Figure 3. B-scan ultrasonography demonstrated a thickened sclera with sub-tenon fluid collection posteriorly (positive T-sign), as shown in Figure 4. Her physical examination was unremarkable, with a normal general appearance, absence of skin rashes, no palpable lymph nodes, no masses in the abdomen, no joint abnormalities or tenderness, no oral ulcers, and normal cardiovascular and respiratory findings.

Peripheral blood film showed hypochromic microcytic anaemia, with a haemoglobin level of 10g/dL. The mean corpuscular volume (MCV) was 64.2 fL (normal range: 77 to 95), and the mean corpuscular haemoglobin (MCH) was 17.3 pg (normal range: 27 to 32). The erythrocyte sedimentation rate was 44 mm/hour (high for age). Her total white cell count was 8.63 x 10^3^/uL, with absolute lymphocyte and neutrophil counts within normal ranges at 2.65 x 10^3^/uL and 5.35 x 10^3^/uL, respectively. Chest X-ray was normal. The results of the infective blood serology screening, which included the Mantoux test, Syphilis, Hepatitis B and C, and Human Immunodeficiency Virus (HIV) tests were negative. Her antinuclear antibody (ANA) tested positive with a titer of 1:1280, showing a homogenous pattern using the indirect immunofluorescence (IIF) method. Further immunology tests of perinuclear staining ANCA (p-ANCA) and diffuse cytoplasmic ANCA (c-ANCA) were negative, however the atypical p-ANCA was positive. Other autoimmune blood screenings including extractable nuclear antigen (ENA) antibody (anti-double-stranded DNA (ds-DNA), anti-Ro, anti-Smith, anti-La, anti-Jo), rheumatoid factor, anti-cyclic citrullinated peptide, antiphospholipid antibodies, myeloperoxidase (MPO) p-ANCA, proteinase 3 (PR3) c-ANCA and glomerular basement membrane antibody were tested negative. Additionally, her complement levels (C3 and C4) were within normal range.

**Figure 5:**
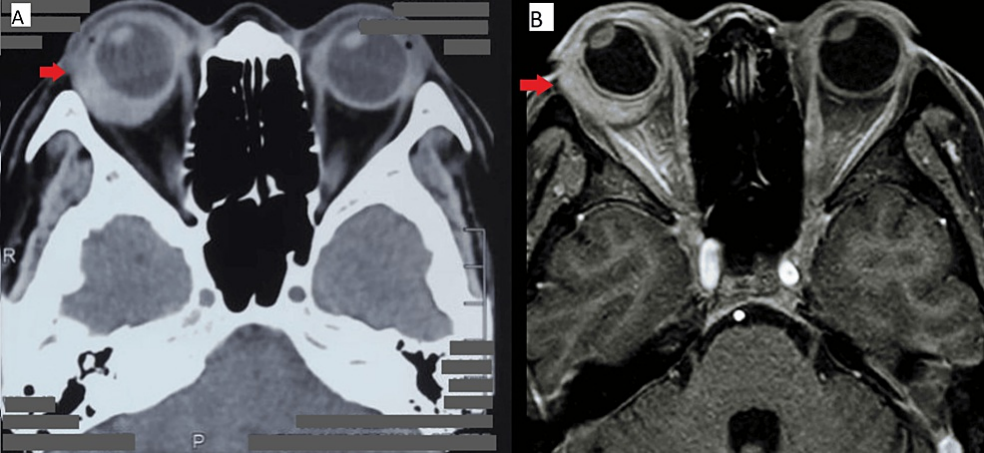
Contrasted computed tomographic scan and magnetic resonance imaging of the brain and orbit demonstrating enhancing hyperdense lesion at right lateral wall measuring 2.0 x 0.5 x 1.8 cm, highlighted with red arrows. 5A: Contrasted computed tomographic scan of the brain and orbit 5B: Magnetic resonance imaging of the brain and orbit

**Figure 6:**
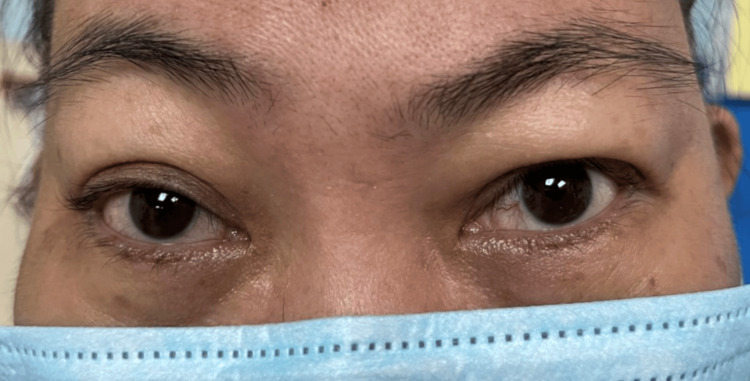
Resolution of right eye proptosis, improvement of bilateral periorbital swelling and redness with improvement of visual functions. The picture was taken post-sixth cycle intravenous cyclophosphamide.

**Figure 7:**
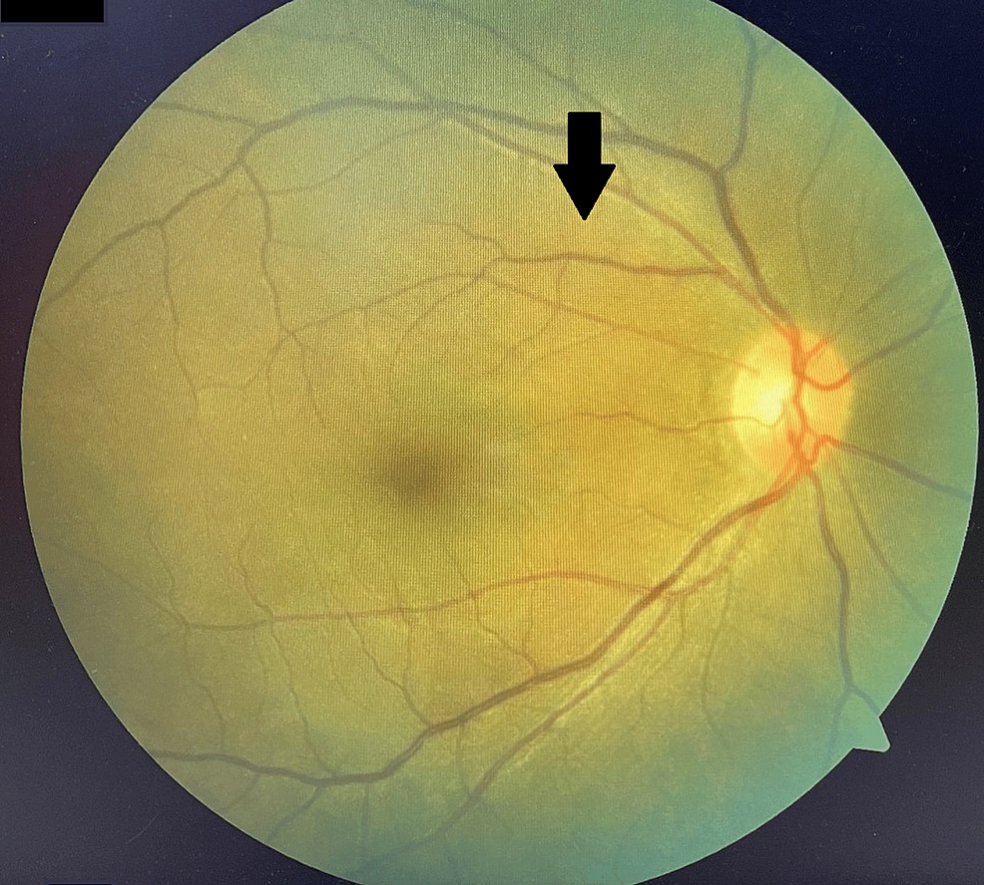
Right eye fundus demonstrating resolution of choroidal folds highlighted with the black arrow.

A contrasted computed tomographic scan of the brain and orbit demonstrated an enhancing hyperdense lesion at the right lateral wall measuring 2.0 x 0.5 x 1.8 cm, as demonstrated in Figure 5. Right eye examination under anesthesia revealed thickened tenon and histopathology examination of the tenon incisional biopsy reported as fibrocollagenous tissue with patchy lymphoplasmacytic infiltration with no obvious granuloma, dysplasia, or evidence of malignancy. She was diagnosed with right eye posterior scleritis secondary to lupus spectrum disease (LSD). The diagnosis of LSD was made based on clinical judgment by the rheumatologist, as the patient did not meet the criteria for typical systemic lupus erythematosus (SLE) classification. However, there was a high index of suspicion due to positive laboratory markers such as antinuclear antibody (ANA) and atypical p-ANCA, coupled with ocular manifestations and a strong family history. She was initially treated with high-dose systemic corticosteroids, including intravenous methylprednisolone 1 gram daily for three days, followed by an oral prednisolone tapering regimen starting at 1 mg/kg (40 mg) for two weeks, then gradually reducing by 2.5 mg to 5 mg every two weeks until reaching a maintenance dose of 5 mg once daily over a duration of six months. She required a topical corticosteroid (prednisolone acetate 1%) administered every 4 hours and four topical ocular hypotensive agents, including timolol 0.5%, latanoprost 0.05%, dorzolamide hydrochloride 2%, and brimonidine tartrate 0.2% to control the intraocular pressure. To further control her disease, the rheumatologist initiated weekly intravenous cyclophosphamide at a dose of 800 mg for six cycles. Additionally, oral hydroxychloroquine at a dose of 200 mg once daily was prescribed. After six weeks following the sixth cycle of intravenous cyclophosphamide, her right vision improved to 6/9 pinhole 6.75, and left vision to 6/7.5. Clinically, the right eye scleritis had completely subsided with full-resolution chemosis, conjunctival injection, and choroidal folds, as shown in Figure 6 and Figure 7. Her extraocular movement also improved with the resolution of diplopia. The posterior sub-tenon fluid collection had also improved on B-scan. Subsequently, her disease was controlled with oral hydroxychloroquine 200 mg once daily, oral mycophenolate mofetil 1000 mg twice daily, and oral prednisolone 5 mg once daily. During follow-up at nine months and one year, the patient's condition remained stable, and there were no further recurrences.

## Discussion

Scleritis is a chronic inflammatory condition that is characterized by edema and cellular infiltration of the sclera and episcleral tissue due to vasculitis of vessels supplying the coat of the eye [1,2]. It is diagnosed based on clinical findings of inflammation and edema affecting sclera tissue with the involvement of superficial and deep episcleral vascularization [3]. The most common classification of scleritis is anterior and posterior. Anterior scleritis is visible upon examination of the anterior eye, whereas posterior scleritis is not visible directly and would require additional radiographical tests such as brightness ultrasonography, computed tomography or magnetic resonance imaging of the orbit to look for posterior eye wall thickening or presence of retrobulbar fluid, producing characteristic T sign [1,3]. If left untreated, the inflammation may progress and spread to adjacent ocular structures resulting in ischemia and necrosis that could cause severe visual impairment and blindness [3].

Scleritis can occur in isolation or as a manifestation associated with systemic vasculitis, where nearly 50% of scleritis patients are associated with immune-mediated diseases [3]. Scleritis is commonly associated with systemic vasculitis diseases that affect small to medium-sized vessels, including RA, GPA, SLE, and inflammatory bowel disease [4]. Conversely, systemic vasculitis diseases that primarily affect large-sized vessels, such as ankylosing spondylitis and giant cell arteritis less commonly result in scleritis [4]. GPA continues to be the primary and common cause of necrotizing scleritis [7]. Whereas, the presence of SLE in patients with scleritis indicates a favorable ocular prognosis as necrotizing scleritis is rare in these cases [4].

Scleritis cases that test positive for p-ANCA or c-ANCA are strongly associated with underlying primary vasculitis disease. Moreover, patients with ANCA-related scleritis tend to experience a higher risk of visual impairment compared to those testing negative for ANCA [3]. Therefore, it is imperative to perform ANCA testing to identify these specific subsets of patients given their heightened risk of developing more severe ocular disease and potentially life-threatening complications, which may necessitate immunosuppressive treatment [3]. ANCA refers to a group of immunoglobulin G (IgG) class autoantibodies, which react against neutrophils and monocytes [8]. Studies have shown ANCA to possess high specificity (98%), high sensitivity (81%), and high negative predictive value (99%), and thus a useful disease marker for small vessel vasculitis [8]. ANCA can be identified by three primary staining patterns: a diffuse cytoplasmic pattern (c-ANCA), a perinuclear pattern (p-ANCA), and an ambiguous staining pattern known as an atypical pattern (atypical ANCA, atypical P-ANCA, X-ANCA). Each subtype of ANCA was associated with different prognostic values, with p-ANCA and c-ANCA generally indicating poorer prognosis and outcomes compared to atypical p-ANCA patterns [8]. c-ANCA are antibodies that detect neutrophil serine proteinase and are seen frequently in patients with GPA, whereas p-ANCA are antibodies that detect lysosomal enzymes myeloperoxidase and are seen mostly in patients with MPA and EGPA [9]. However, these associations are not exclusive and may occur interchangeably.

Atypical p-ANCA pattern is rare and can react with a variety of antigens, leading to its association with various diseases [10]. Due to its lack of specificity, it is not considered a reliable serum marker for vasculitis [8]. Atypical p-ANCA can be further classified into type A and type B. Type A atypical p-ANCA exhibits broad inhomogeneous perinuclear staining of neutrophils, while type B atypical p-ANCA shows a mix of broad inhomogeneous perinuclear and diffuse granular cytoplasmic staining patterns. Atypical p-ANCA has been reported in autoimmune diseases such as RA, SLE, inflammatory bowel disease, and primary sclerosing cholangitis [8]. About 10% of patients with ocular inflammatory diseases (OID) test positive for atypical p-ANCA [8]. Additionally, positive atypical p-ANCA may result from the use of propylthiouracil and other drugs even in the absence of vasculitis [11]. Reports have also linked positive atypical p-ANCA with chronic suppurative lung disease, infection, malignancies, and renal disorders [8].

The most common ocular manifestation reported in patients with atypical p-ANCA was uveitis (up to 60%), followed by scleritis (20%), ocular cicatricial pemphigoid (9%), dry eyes (6%), and conjunctivitis (3%) [8]. The presence of white matter lesions in the central nervous system of patients with atypical p-ANCA suggests its potential involvement in optic neuritis among patients with multiple sclerosis [8]. Compared to other forms of ANCA, the majority of the patients with atypical p-ANCA do not have vasculitis disease and demonstrated favourable outcomes with effective disease control using immunosuppressive therapy [8]. This further highlights the better ocular prognosis in patients with atypical p-ANCA.

Lupus spectrum disease (LSD) encompasses a range of symptoms that are incomplete to fulfill the criteria for a diagnosis of SLE. The diagnosis of LSD relies heavily on clinician suspicion of an experienced clinician, by taking into account factors such as symptoms present in at least one organ system, positivity of antinuclear antibody (ANA), and the clinical suspicion of future development of SLE [6]. Under the umbrella term of LSD, it includes conditions such as lupus nephritis, cutaneous lupus erythematosus (CLS), antiphospholipid antibody syndrome, ANA positive syndrome, and secondary Sjogren syndrome [12]. Due to the extensive literature available on SLE and its ocular manifestation compared to the lack of information on ocular involvement in LSD, SLE will be the primary reference used to describe ocular condition in LSD.

SLE is a complex autoimmune disease that is associated with a wide range of autoantibodies [13]. The classical examples of autoantibodies in SLE include anti-double stranded (ds) DNA, anti-Smith, and anti-histone antibodies among many others, however, several studies have also reported the presence of ANCA in SLE [5,14]. The prevalence of ANCA in SLE patients ranges from 25%-56% [5]. In one study involving 84 SLE patients, 9.5% tested positive for p-ANCA and 31% tested positive for atypical ANCA [14]. Another study supported these findings, with atypical ANCA being the most predominant variant in SLE patients [5]. However, another study noted p-ANCA to be the predominant pattern in SLE patients, with up to 93% of SLE patients testing positive for p-ANCA, including those with drug-induced lupus [5].

Ocular manifestations are prevalent in SLE, occurring in about one-third of SLE patients [15]. Ocular manifestations in SLE encompass a spectrum of conditions, including keratoconjunctivitis sicca (25%), corneal involvement presenting as recurrent corneal erosions, peripheral ulcerative keratitis, interstitial keratitis, episcleritis, and scleritis (1%). Additionally, lupus retinopathy (10%) and optic nerve involvement (1%) are observed. While rare, orbital involvement presenting as orbital vasculitis and/or myositis may occur, with clinical symptoms such as proptosis, blurred vision, chemosis, and restricted eye movement and scans revealing extraocular muscle enlargement [15]. Posterior scleritis is rarely seen in patients with SLE [15]. Our case has highlighted a rare case of posterior scleritis in a patient with underlying LSD. Instead of meeting the typical diagnostic criteria of SLE as described by the American College of Rheumatology 2019 criteria, our case was carefully evaluated by a rheumatologist and was determined to be LSD, supported by positive ANA and ocular manifestations. This diagnosis was further supported by a strong clinical suspicion given the patient's family history of SLE and the presence of atypical p-ANCA, which literature indicates can be found in 31% of SLE cases [14].

In approaching a case of scleritis, a high index of clinical suspicion is crucial, involving a comprehensive assessment for any relevant non-ocular history and/or symptoms suggestive of the diagnosis. Complete blood counts, including hemoglobin, platelet, and white cell count as well as inflammatory markers such as C-reactive protein (CRP), and erythrocyte sedimentation rate (ESR) are valuable to indicate the presence of an inflammatory process. Moreover, autoimmune blood markers such as antinuclear antibody (ANA), extractable nuclear antigen (anti-ds-DNA, anti-histone, anti-Smith, anti-Ro, anti-La, anti-Jo), rheumatoid factor (RF), complement levels (C3 and C4), antiphospholipid antibodies, vasculitis autoimmune profile (c-ANCA, p-ANCA, atypical c-ANCA, atypical p-ANCA, myeloperoxidase p-ANCA, proteinase 3 c-ANCA, glomerular basement membrane antibody), are essential in establishing a diagnosis of immune-mediated systemic vasculitis related to scleritis.

Treatment of scleritis typically involves systemic therapies, especially for moderate to severe cases. Mild to moderate anterior scleritis is often managed with oral nonsteroidal anti-inflammatory drugs (NSAIDs) alone or combined with topical corticosteroids like prednisolone acetate or dexamethasone [1]. However, if initial therapies fail, more aggressive options such as systemic corticosteroids (oral or intravenous) may be necessary. Immunosuppressive therapy is reserved for patients with necrotizing scleritis or severe, complicated scleritis unresponsive to corticosteroids. In the treatment of scleritis related to SLE, especially in cases of severe ocular involvement such as scleritis or orbital inflammation, immediate systemic treatment is important [15]. Typically, intravenous pulsed steroid therapy (methylprednisolone 1g/day for three to six days is initiated) then followed by oral prednisolone [16]. If steroid treatment fails to achieve the desired effect, immunosuppressive agents such as methotrexate, mycophenolate mofetil, azathioprine, and cyclophosphamide may be considered [16]. In SLE patients with scleritis, antimalarial drugs like hydroxychloroquine are also often used as adjunctive therapy to control disease activity and reduce the need for higher doses of steroids or immunosuppressants [17]. In our case, the patient was administered intravenous therapy methylprednisolone at 1g/day for three days during the acute phase, followed by a tapering regimen of oral prednisolone over six months and six cycles of intravenous cyclophosphamide. Subsequently, maintenance therapy comprised hydroxychloroquine, mycophenolate mofetil, and oral prednisolone at a daily dose of 5mg. Overall, the patient has responded favourably to the treatment provided.

This case demonstrates a positive outcome in treating atypical p-ANCA posterior scleritis associated with lupus spectrum disease. Employing a multidisciplinary approach to diagnosis and treatment is crucial, as scleritis is a rare manifestation of lupus spectrum disease, posing a diagnostic challenge to clinicians. Timely intervention by both rheumatologists and ophthalmologists would significantly reduce patient mortality and morbidity, as shown in this case.

## Conclusions

This case demonstrates a positive outcome in treating atypical p-ANCA posterior scleritis associated with lupus spectrum disease. Employing a multidisciplinary approach to diagnosis and treatment is crucial, as scleritis is a rare manifestation of lupus spectrum disease, posing a diagnostic challenge to clinicians. Timely intervention by both rheumatologists and ophthalmologists would significantly reduce patient mortality and morbidity, as shown in this case.
